# Individual Physician versus Team-Based Medical Encounters: Maximizing the Efficiency of a Medical Relief Service in Rural Honduras

**DOI:** 10.1155/2011/852963

**Published:** 2011-07-05

**Authors:** Rachel Whitney, Michael Stevens, Gonzalo Bearman

**Affiliations:** ^1^Division of Internal Medicine, Department of Infectious Disease, School of Medicine, Virginia Commonwealth University, Richmond, VA 23298, USA; ^2^Division of Internal Medicine, Department of Infectious Disease, Virginia Commonwealth University, Richmond, VA 23298, USA

## Abstract

*Background*. For several years, the Virginia Commonwealth University (VCU) Department of Internal Medicine has traveled to the towns of La Hicaca and Coyoles in rural Honduras. In 2010, a new encounter method was employed during the brigade in Coyoles. *Objectives*. To examine the differences in clinical encounters and adherence with chronic diseases and public health screening between the traditional and team-based encounter methods. *Methods*. Chi-square analysis was used to determine statistical significance between encounter methods over several variables used in the standard interview form. *Results*. 314 patients were interviewed using the team-based approach, and 153 patients were interviewed with the traditional model. Statistically significant increases in compliance using the team-based method were observed with diabetes screening and selecting candidacy for antihelminthic therapy. Other variables with significantly increased compliance using the team-based method were compliance with checking a blood glucose value, diagnosing GERD, and prescribing medication such as analgesics and multivitamins. *Conclusion*. Our results show a statistically significant increase in compliance with data collection and clinical screening using the new team-based encounter method. This design provides a more goal-oriented approach to the patient interview. These data will help guide more effective delivery of health care on future medical relief trips to Honduras.

## 1. Introduction

Short-term medical relief trips from the United States to destinations in developing countries are common, with an estimated hundreds of trips traveling from the United States yearly [1]. These relief trips suffer from a number of limitations. Often volunteer providers have little experience working in such a resource-deprived environment, they have a poor grasp on what is or is not appropriate in terms of care, and many do not return after the initial trip. Other common failings of these trips come from poor assessment and management of chronic illnesses such as diabetes and hypertension. We discuss a novel clinical encounter mechanism designed to improve the care we provide on a regular short-term medical relief trip to Northern Honduras. 

Since 2006, the Virginia Commonwealth University (VCU) Department of Internal Medicine has been sending a team of internists to rural Honduras to provide medical services, largely emphasizing the treatment of acute illnesses. Since 2008, our group has focused its efforts on two communities in the Department of Yoro area of Northern Honduras; a remote, mountainous area in and around the town of La Hicaca, and several communities near the more developed city of Olanchito (Coyoles). 

Our group created the Adult Health Initiative (AHI) in 2009 to help optimize and standardize the care provided by the relief team, as well as to begin addressing chronic illness [2, 3]. A novel clinical encounter form was developed that employed multiple algorithms, including screening for diabetes, assessing candidacy for antihelminthic therapy and prenatal vitamins, and chronic conditions such as gastrointestinal illnesses and musculoskeletal concerns. Coupled with this new clinical encounter form, we also developed a novel clinical encounter mechanism. On our June 2010 relief trip, the new encounter form and clinical encounter mechanism were employed. 

**Figure 1:**
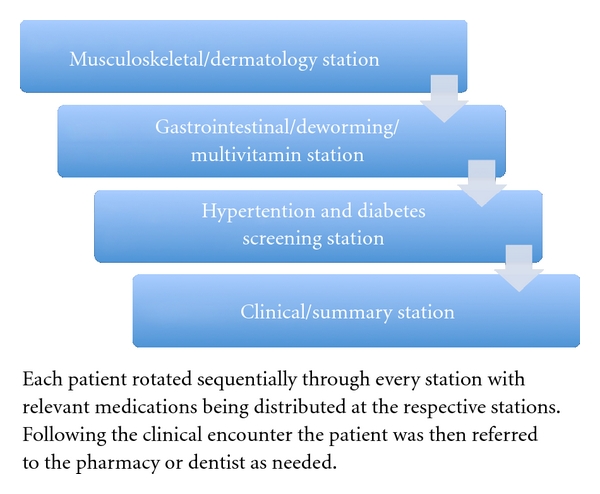
Team-based encounter mechanism.

**Figure 2:**
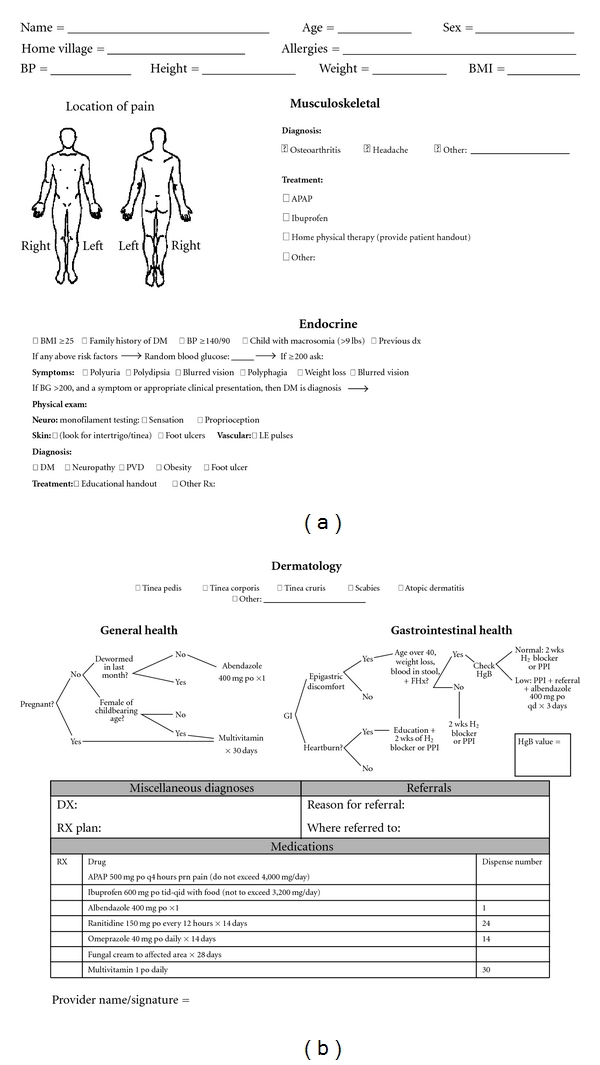
Clinical encounter form.

During the first part of the June 2010 brigade (in the area in and around La Hicaca) the traditional “one patient one provider” encounter mechanism was used. Under the individual provider system, each physician was responsible for addressing all of the elements on the clinical encounter form, including assessment for diabetes, osteoarthritis, gastrointestinal illness, and candidacy for antihelminthic therapy and multivitamins. In Coyoles, the new “team-based” encounter mechanism was employed. With the team-based approach, four stations were established (Figure 1), through which each patient progressed sequentially. A physician and medical student were present at each station. The encounter mechanism included a musculoskeletal/dermatology station, a gastrointestinal/multivitamin station, a hypertension/diabetes screening station, and lastly the clinician/summary station. This last station addressed any remaining clinical or dental problems not previously addressed by the other stations. At both sites the same clinical encounter form was utilized (Figure 2). In this study, we examine the utility of the novel team-based encounter mechanism and compare it to the traditional one patient to one provider model with a focus on data collection and clinical encounter screening. 

## 2. Objectives

We examined the effectiveness of the traditional versus the team-based clinical encounter methods. Outcomes of interest included provider compliance with screening for diabetes, gastrointestinal and dermatologic conditions, along with assessment for candidacy of antihelminthics and multivitamins. We also examined differences in other measures of care, including the proportion of patients diagnosed with osteoarthritis, gastrointestinal illnesses, diabetes and physical therapy education, and medications prescribed.

## 3. Methods

 Two clinical encounter mechanisms, the “traditional” one patient to one physician model versus the “team-based” encounter method were compared, respectively, between La Hicaca and Coyoles (Table 1). All stations were attended by a physician. Patients at Coyoles who were not seen via the team-based clinical encounter method were excluded (24 patients); this yielded a total of 467 patients. Chi-square tests of independence using the Predictive Analytics Software (PASW) 18 statistics software package for MAC were performed to examine for differences between the traditional and team-based encounter methods for the following data: compliance with antihelminthic therapy candidacy, and adherence with the algorithmic provision of multivitamins to women of childbearing age (18–51 yrs). Diabetes screening was assessed on two levels. The first was provider assessment of predetermined patient risk factors as specified in the encounter form. The second was performance of a blood glucose check by finger stick when risk factor analysis suggested increased risk of diabetes. Additionally, the Chi-square test for statistical significance was performed to compare the differences in the following: diagnosis of osteoarthritis, diagnoses of dyspepsia and GERD, analgesics prescribed, acetaminophen prescribed, ibuprofen prescriptions, H2-blockers or proton pump inhibitors (H2/PPI) prescribed, total multivitamins (MVI) prescribed (not just in women of childbearing age), topical antifungals prescribed, diabetes education handouts given, and home physical therapy handouts given. Fisher's exact test was used for all variables with low frequency counts (<5).

## 4. Results

There were 338 patients interviewed at the Coyoles site, and 153 patients interviewed at La Hicaca. Twenty-four patients at Coyoles were not interviewed using the team-approach, and so they were excluded from the method analysis. This left a total of 314 patients interviewed and treated using the team-based approach, and 153 patients interviewed using the traditional method (Table 1). 

The team-based encounter mechanism resulted in increased adherence with clinical evaluation elements. For diabetes screening, Chi-square analysis revealed a significant difference between methods, with 311 (99%) patients screened with the team-based method and 61 (39%) patients screened with the traditional method, *P* = 0.000. For blood glucose value checked, 132 (42%) patients were tested with the team-based approach and 9 (5.9%) patients were tested with the traditional method, *P* = 0.000. Compliance with the antihelminthic therapy candidacy screening was also significantly different between the two encounter mechanisms, with 304 (96.8%) patients screened with the team-based approach and 139 (90.8%) patients screened with the traditional mechanism, *P* = 0.006. For the diagnosis of GERD, a significant difference between encounter methods was observed, with 191 (60.8%) patients screened with the team approach and 34 (22.2%) screened with the traditional approach, *P* = 0.000. A statistically significant difference was observed between encounter methods for the number of drug prescriptions written. There were 287 (91.4%) prescriptions written for analgesics using the team approach, and 123 (80.4%) written using the traditional model, *P* = 0.001. There were 263 (83.3%) acetaminophen prescriptions written using the team approach, and 86 (56.2%) written with the traditional model, *P* = 0.000. There were 55 (17.5%) prescriptions written for ibuprophen using the team model, and 40 (26.1%) written using the traditional model, *P* = 0.030. For H2-blockers and PPIs, 152 (48.4%) prescriptions were written via the team-based approach, and 44 (28.8%) prescriptions were written via the traditional model, *P* = 0.000. Lastly, there was a significant difference between the number of multivitamin prescriptions written using the team approach versus the traditional approach, with 297 (94.6%) written using the team model and 120 (78.4%) written using the traditional model. 

No statistically significant difference was observed for compliance with diabetes education, with 14 (4.5%) patients receiving a diabetes education handout through the team model and 2 (1.3%) patients receiving diabetes education handouts through the traditional approach, *P* = 0.104. For the remaining variables, no significant differences were observed between the two models. There were 72 (22.9%) patients diagnosed with osteoarthritis via the team model, and 27 (17.6%) patients diagnosed via the traditional model, *P* = 0.190. There were 23 (7.3%) patients diagnosed with dyspepsia using the team-based approach, and 14 (9.2%) patients diagnosed using the traditional approach, *P* = 0.386. There were 52 (16.6%) patients prescribed topical antifungal cream via the team model, and 15 (9.8%) patients who received a prescription for the cream using the traditional model, *P* = 0.051. There were 29 (9.2%) patients given a home physical therapy (PT) handout via the team model, and 12 (7.8%) patients given the handout via the traditional model, *P* = 0.618. Of the 491 total patients, there were 273 women aged 18–51. For women of childbearing age, 150 (87.2%) received a multivitamin prescription via the team-based approach while 93 (92.1%) patients of this group received a multivitamin prescription via the traditional model, *P* = 0.214.

## 5. Discussion

Medical relief work in developing countries is common. The VCU Honduras program consists of a team of physicians, residents, students, and pharmacists. Over the years, the VCU Honduras relief effort has provided direct medical care for acute and chronic conditions to the same rural Honduran communities [4]. In addition to the management of acute and chronic conditions, the team's public health mission focuses on providing antihelminthic therapy to both adults and children. The World Health Organization (WHO) has identified Honduras as a high-prevalence area for soil-transmitted helminthes, with an estimated prevalence of ≥50% [5]. These infections profoundly contribute to the populations' morbidity and are a significant cause of mortality as well [6]. 

The AHI project was designed to help lay the groundwork to better assess and address other noninfectious chronic illnesses of public health significance, including diabetes, hypertension, and obesity. The Pan American Health Organization's report on Health in the Americas stated that 36% of the adult population of Tegucigalpa was overweight and another 23% were obese. This same report found the prevalence of diabetes in this population at 7.8%, nearly half of whom were unaware of their diagnosis. In 2004 the incidence of diabetes was 593 per 100,000 people [7]. The utilization of the novel clinical encounter form and the team-based clinical encounter mechanism allowed us to better document the prevalence of diabetes in our clinic population and to improve our ability to perform diabetes screening. 

Recognizing that care across providers on relief trips is often inconsistent, the novel team-based encounter mechanism was developed in an attempt to increase physician uniformity and adherence to health screening as specified in the encounter form. The traditional clinical encounter mechanism involves one practitioner completing the entire form with each patient. The team-based approach divides the encounter across four separate stations and providers. We believe that this method increases compliance by creating a more focused, goal-oriented encounter at each station, allowing for better, less fragmented and more thorough patient care. 

Based on our experience, when different providers are expected to complete an entire encounter form per patient, discrepancies in information obtained and varying levels of completeness are observed. Any number of factors can affect this, from communication difficulties with the patient to provider fatigue. Incomplete data hinders our efforts to best tailor our medical relief program to the public health needs of the population. 

We documented a significant improvement in compliance with many aspects of patient evaluation, favoring the team-based encounter mechanism over the traditional method. Under a team-based encounter approach, screening for diabetes, osteoarthritis, dermatologic conditions, and antihelminthic therapy was more successful. These were all performed prior to the final clinical and summary encounter with a physician. This format also led to increased frequency of GERD diagnosis, and a greater number of medication prescriptions written for analgesics and MVIs.

A potential disadvantage of the new team-based method is the loss of continuity between patient and provider during a single encounter. By rotating through stations, the patient meets with a minimum of four different physicians during one encounter. This system could disrupt the flow of the visit and be uncomfortable or distracting for the patient. It may also give the impression that patients are being processed through the medical equivalence of an assembly line. In this way the traditional approach is better at supporting patient-provider continuity during an encounter.

A total of 338 patients were examined at Coyoles, and 153 patients were examined in La Hicaca. On average, 84 patients were interviewed per day over the 4 days in Coyoles while 51 patients were interviewed per day in La Hicaca over 3 days. We believe that the team-based encounter mechanism did not adversely affect patient throughput during the clinic sessions. Although subject to a standardized, more structured approach, the stations likely allowed for quicker and more focused data collection without compromising clinical care and face-to-face time with a physician. 

The utilization of a structured, team-based clinical encounter mechanism allowed us to more thoroughly and methodically assess both acute and chronic illnesses of public health concern, such as antihelminthic candidacy screening, obesity, diabetes, dermatologic infections, and osteoarthritis. This approach is of benefit to the patients as the spectrum of their medical concerns is fully addressed during the course of a structured, station-based encounter.

There is a paucity of data on the optimal clinical encounter mechanism for medical relief trips to developing countries, and this study adds to the small body of literature as an example of how to better structure a medical relief brigade in this environment.

Our study has several strengths. The same team of physicians traveled to both sites, allowing for consistent interview personnel regardless of which approach was used. Also, the data used to compare methods was collected on the same medical relief trip, eliminating change in population over time as a confounder. A major limitation of our study is that patients were not randomized to either encounter method group. Rather, the traditional model and the team-based models were employed in two different towns. While both are poor and have limited access to healthcare, nuanced differences may exist in demography and health status. La Hicaca, where the traditional method was used, is a rural, mountainous region located 75 kilometers from the nearest city and medical center. However, Coyoles, where the new team-based approach was employed, is in a flatter, more urbanized setting about 15 kilometers from a metropolitan center. It is possible that health differences in these populations may exist owing to different environments and to differing levels of healthcare access. These differences may have affected the disparities in diagnoses and prescription patterns observed between the two encounter methods. Of note, the community with the greatest poverty and isolation (La Hicaca), evaluated by the traditional encounter method, had a smaller proportion of patients with GERD, and received less H2/PPI medications. This may be a reflection of missed diagnostic and therapeutic opportunities by the traditional encounter or a marker of better health status. 

These findings have important implications for future medical relief trips, including a more efficient restructuring of clinical workflow and a better ability to assess and address chronic disease. By better addressing chronic illness and optimizing various aspects of our public health focus, the data from the AHI project will allow us to more effectively address the health needs of select communities in Northern Honduras.

## Figures and Tables

**Table 1:** Comparison of clinical encounter methods.

Variable	Team based *N* = 314	Traditional *N* = 153	Team based versus traditional
Compliance with diabetes screen	311 (99%)	61 (39%)	*P* = 0.0001
Blood glucose value checked	132 (42%)	9 (5.9%)	*P* = 0.0001
Diabetes education handout given	14 (4.5%)	2 (1.3%)	*P = 0.1040
Compliance with antihelminthic therapy candidacy screen	304 (96.8%)	139 (90.8%)	*P* = 0.0060
Diagnosis of osteoarthritis	72 (22.9%)	27 (17.6%)	*P* = 0.1900
Home PT handout given	29 (9.2%)	12 (7.8%)	*P* = 0.6180
Prescribed an analgesic	287 (91.4%)	123 (80.4%)	*P* = 0.0010
Prescribed acetaminophen	263 (83.3%)	86 (56.2%)	*P* = 0.0001
Prescribed ibuprophen	55 (17.5%)	40 (26.1%)	*P* = 0.0300
Diagnosis of dyspepsia	23 (7.3%)	14 (9.2%)	*P* = 0.3860
Diagnosis of GERD	191 (60.8%)	34 (22.2%)	*P* = 0.0001
Prescribed an H2/PPI	152 (48.4)	44 (28.8%)	*P* = 0.0001
Prescribed an MVI	297 (94.6%)	120 (78.4%)	*P* = 0.0001
Women of childbearing age prescribed an MVI	150 (87.2%)	93 (92.1%)	*P* = 0.2140
Prescribed topical antifungals	52 (16.6%)	15 (9.8%)	*P* = 0.0510

*Fisher's exact test.
